# Chipmunk Parvovirus Is Distinct from Members in the Genus *Erythrovirus* of the Family *Parvoviridae*

**DOI:** 10.1371/journal.pone.0015113

**Published:** 2010-12-03

**Authors:** Zhaojun Chen, Aaron Yun Chen, Fang Cheng, Jianming Qiu

**Affiliations:** 1 Department of Clinical Laboratory, The Affiliated Hospital of Hangzhou Normal University, Hangzhou, China; 2 Department of Microbiology, Molecular Genetics and Immunology, University of Kansas Medical Center, Kansas City, Kansas, United States of America; University of Hong Kong, People's Republic of China

## Abstract

The transcription profile of chipmunk parvovirus (ChpPV), a tentative member of the genus *Erythrovirus* in the subfamily *Parvovirinae* of the family *Parvoviridae*, was characterized by transfecting a nearly full-length genome. We found that it is unique from the profiles of human parvovirus B19 and simian parvovirus, the members in the genus *Erythrovirus* so far characterized, in that the small RNA transcripts were not processed for encoding small non-structural proteins. However, like the large non-structural protein NS1 of the human parvovirus B19, the ChpPV NS1 is a potent inducer of apoptosis. Further phylogenetic analysis of ChpPV with other parvoviruses in the subfamily *Parvovirinae* indicates that ChpPV is distinct from the members in genus *Erythrovirus*. Thus, we conclude that ChpPV may represent a new genus in the family *Parvoviridae*.

## Introduction

Parvoviruses, members of family *Parvoviridae*, are small, non-enveloped icosahedral viruses. They contain a linear, single-stranded DNA genome with terminal hairpin structures at both ends. Parvoviruses that infect vertebrates and non-vertebrates are classified as subfamilies *Parvovirinae* and *Densovirinae*, respectively. There are five genera in the subfamily *Parvovirinae*: *Parvovirus*, *Dependovirus*, *Erythrovirus*, *Bocavirus* and *Amdovirus* [1], [2]. In addition to human parvovirus B19 (B19V) and non-human primate *Erythrovirus* [simian parvovirus (SPV) [3], pig-tailed macaque parvovirus (PtMPV), and rhesus macaque parvovirus (RhMPV) [4]], the genus *Erythrovirus* has two tentative members, the bovine parvovirus type-3 (BPV3) [5] and the chipmunk parvovirus (ChpPV) [6]. These two tentative members are classified as such based mainly on their sequence similarities to other members. ChpPV was initially isolated from Hepatitis B surface antigen (HBsAg)-positive sera from Manchurian chipmunks in Korea, and its genome is 47% identical to that of the prototype B19V [6]. The putative capsid protein shows a homology of over 34% with those of the B19V and SPV, but only less than 20% with those of other parvoviruses [6]. Little is known about the molecular features of this virus. The virus has not been isolated, and an infectious clone has not been established.

The large non-structural protein (NS1) of parvoviruses is a multifunctional viral nonstructural protein, which contains DNA binding, ATPase, helicase and nuclease activity and is essential for viral DNA replication [7]. The NS1 of parvoviruses in the genus *Erythrovirus* and *Parvovirus* also has been shown to induce cell death, which is critical to their pathogenesis [8]–[11]. However, the *Bocavirus* NS1 *per se* does not induce cell death [12]. The B19V NS1 induces apoptosis in both B19V permissive and non-permissive cells [8], [13], [14]. The amino acid sequence of the ChpPV NS1 diverges from that of the prototype B19V NS1 by 74.2% [6]. The potency of this novel parvovirus NS1 protein in inducing apoptosis has not yet been examined.

In the current study, we have used a replication-competent system in COS-7 cells to systematically characterize the transcription profile of ChpPV. Finally, we evaluated the potency of the novel ChpPV NS1 protein in inducing apoptosis in B19V semi-permissive UT7/Epo-S1 cells [15].

## Materials and Methods

### Cells

COS-7 cells (CRL-1651; ATCC) were maintained in Dulbecco's modified Eagle's medium (DMEM) with 10% fetal calf serum (FCS) at 37°C in 5% CO_2_. UT7/Epo-S1 cells were cultured in DMEM with 10% FCS and 2 units/ml of Epo at 37°C in 5% CO_2_ as previously described [14], [16].

### Transfection

Two µg of DNA was transfected into COS-7 cells on one 60-mm dish, using Lipofectamine and Plus reagents (Invitrogen) as previously described [17]. UT7/Epo-S1 cells were transfected with 2 µg of DNA per 2×10^6^ cells in a universal electroporation reagent using the Nucleofector (Lonza, MD) as previously described [17].

### Plasmid construction

#### (i) pC1ChpPV plasmid

A pCR II (Invitrogen) based plasmid containing the genome of ChpPV without the left and right ends was a gift from Dr. Byung Chui Yoo at Chung Ang University Hospital, Korea. This pCRIIChipPV plasmid has an extra sequence of 109 nts in front of the published ChipPV sequence (Genbank accession no.: U86868). The whole ChpPV sequence (nt 1–5,205) composed of a functional promoter region, was deposited at Genbank as accession no. GQ200736. The pC1ChpPV was constructed by replacing the B19V *NSCap* gene with the ChpPV sequence of nt 1–5,205 in pC1NS1(−) [16], [18].

#### (ii) GFP-fused NS1 construct

The NS1 ORFs of ChpPV (nt 306–2438) and B19V (nt 616–2628, GenBank: AY386330.1) were inserted into BamHI-XhoI digested pcDNAGFP_C3HA to construct the pGFP-ChpPVNS1HA and pGFP-B19VNS1HA, respectively. The pcDNAGFP_C3HA was constructed by inserting a 3 × HA-encoding sequence between XhoI and ApaI sites in pcDNAGFP [16].

#### (iii) Clones used to generate probes for RNase protection assays

To map the transcription units of ChpPV, we used ChpPV P1, P2, P3, P4, P5, P6 and P7. These probes were constructed by cloning the following regions of ChpPV into BamHI-HindIII digested pGEM4Z (Promega): nt 110–318 (ChpPV P1), nt 2088–2488 (ChpPV P2), nt 2269–2588 (ChpPV P3), nt 2669–2988 (ChpPV P4), nt 3092–3331 (ChpPV P5), nt 4909–5205 (ChpPV P6) and nt 1761–2000 (ChpPV P7).

### RNA isolation and RNase protection

Total RNA was isolated from transfected cells 2 days posttransfection using TRIZOL reagent (Invitrogen). Probes were generated from BamHI-digested templates by *in vitro* transcription with T7 polymerase using the MAXIscript® kit (Ambion) and following the manufacturer's instructions. RNase protection assays were performed essentially as previously described [19], [20]. Signals of RNase protection were quantified with the Storm 856 phosphor imager and Image Quant TL software v2005 (GE Healthcare). Relative molar ratios of individual RNA species were determined after adjusting for the number of ^32^P-labeled uridines (U) in each protected fragment as previously described [20].

### Northern and Southern blotting analysis

Five µg of total RNA was used for Northern blotting analysis as described previously [21], [22]. Blots were hybridized with the ChpPV *NS*, *Cap and NSCap* probes, which were amplified ChpPV DNA fragments of nt 1–1561, nt 3281–5205 and nt 1–5205, respectively, from the pC1ChpPV. Low-molecular-weight DNA (Hirt DNA) was extracted from pC1ChpPV-transfected COS-7 cells, and DpnI digestion and Southern blotting were performed as described previously [23] using the ChpPV *NSCap* probe. Signals were developed by exposing the blots to X-ray films.

### Western blotting analysis

SDS-PAGE and Western blotting were performed essentially as described previously [23]–[25].

### Flow cytometry analysis

Cells were double-stained live with Cy5-conjugated AnnexinV (BD Biosciences) and Propidium Iodide (PI, Sigma) according to the manufacturer's instructions (BD Biosciences). All samples were analyzed on the three-laser flow cytometer (LSR II, BD Biosciences) at the Flow Cytometry Core of the University Kansas Medical Center. Flow cytometry data were analyzed using FACS DIVA software (BD Biosciences).

## Results

### The transcription profile of ChpPV is unique from those of the members in the genus *Erythrovirus*

**Figure 1:**
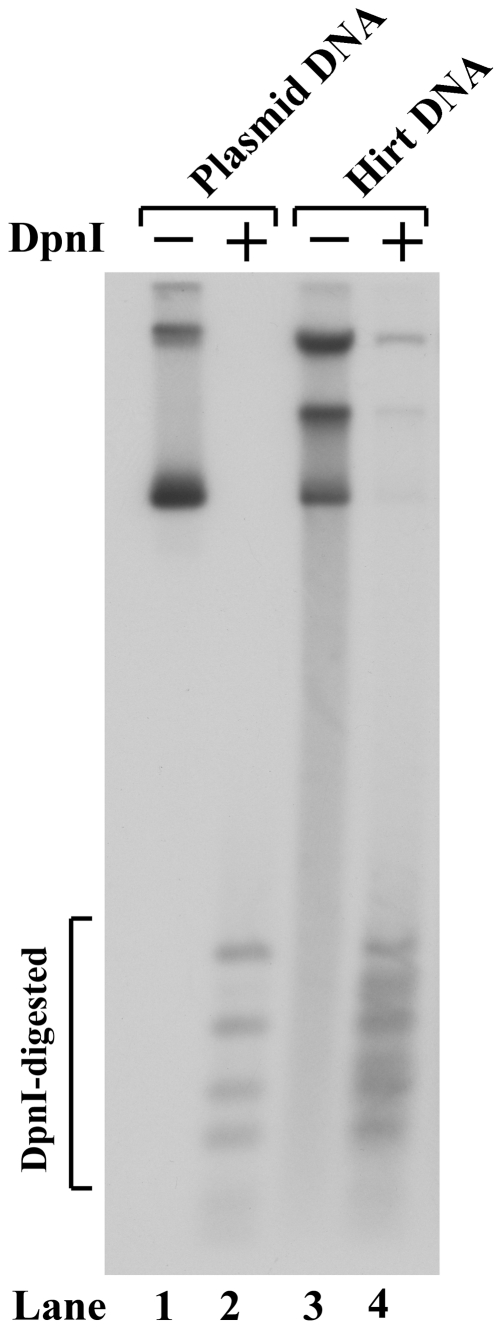
Replication of the construct pC1ChpPV in COS-7 cells. COS-7 cells were transfected with pC1ChpPV. Hirt DNA sample was extracted from the cells at 48 hrs post transfection, and were digested with DpnI (DpnI +) *vs.* without DpnI (DpnI -). Samples were subjected to Southern blotting analysis using the ChpPV *NSCap* probe (nt 1–5205). Ten ng of pC1ChpPV plasmid DNA were treated in parallel as a DpnI-digestion control (lane 2).

A nearly full-length genome of ChpPV was cloned into an SV40-ori-containing plasmid. Replication of the ChpPV genome on this plasmid in COS-7 cells was confirmed by Southern blotting analysis, as the DpnI digestion-resistant DNA bands are shown (Fig. 1, lane 4). Using this expression-capable ChpPV genome, we obtained a detailed transcription profile of ChpPV, which was generated following transfection of COS-7 cells.

### RNase protection assays of ChpPV RNA

**Figure 2:**
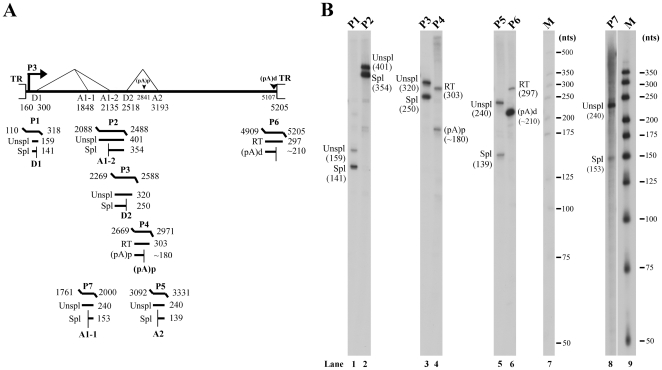
Transcription mapping of ChpPV RNA by RNase protection assays. (A) **Schematic diagram of the ChpPV genome and the probes used for analysis by** RNase protection assays. The transcription units, with the promoter (P3), splice donor (D1 and D2) and acceptor (A1-1, A1-2, A2) sites, and polyadenylation signals, (pA)p at nt 2841 and (pA)d at nt 5107, are indicated. All the ChpPV probes are shown with their respective nucleotide numbers, along with the designated bands they are expected to protect and their predicted sizes. Spl, spliced RNAs; Unspl, unspliced RNAs; RT, RNAs read-through the (pA)p site. (**B**) **Mapping of the ChpPV transcription units by** RNase protection assays. Ten µg of total RNA isolated two days after the transfection of COS-7 cells with plasmid pC1ChpPV were protected by ChpPV probes P1-P7. Lanes 7&9, ^32^P-labeled RNA markers with sizes indicated.

Total RNA isolated from pC1ChpPV transfected COS-7 cells was subjected to RT-PCR with various primers based on the transcription maps of B19V and SPV [26], [27]. Transcription units identified by RT-PCR were summarized in Fig. 2A, and were used to design probes targeted to the promoter, intron donor and acceptor sites, and polyadenylation sites, for use in RNase protection assays.

### ChpPV P1 probe

The ChpPV P1 probe, which spans the putative P3 promoter (Fig. 2A), was protected to generate two bands of approximately 159 nts and 141 nts, respectively. The 159-nt and 141-nt bands represent RNAs that were transcribed from the P3 promoter and were unspliced and spliced at the first donor site (D1), respectively. Thus, the P3 RNA initiation site was located at approximately nt 160, and the D1 site was confirmed to be at nt 300. The ChpPV RNA spliced from the D1 site accumulated to approximately five times greater levels than the ChpPV RNA that was unspliced at the D1 (Fig. 2B, lane 1).

### ChpPV P2 and P3 probes

The ChpPV P2 probe, which spans the putative A1-2 acceptor, was protected to generate two bands of 401 nts and 354 nts (Fig. 2B, lane 2). The bands at 401-nt and 354-nt represent RNAs unspliced and spliced at the A1-2, respectively. The ChpPV P3 probe, which spans the putative D2 donor, protected two bands of 320 nts and 250 nts (Fig. 2B, lane 3). The bands at 320-nt and 250-nt represent RNAs that were unspliced and spliced at the D2 donor, respectively. The ratio of RNA spliced *vs.* unspliced at the D2 donor site was approximately 1∶1.

### ChpPV P4 and P5 probes

The ChpPV P4 probe, which spans the putative internal polyadenylation site, was protected to yield bands at 303 nts and approximately 180 nts (Fig. 2B, lane 4). The 303-nt and ∼180-nt bands represent RNAs read through and polyadenylated at the (pA)p site, respectively. Apparently, less than half of the RNAs were polyadenylated at the (pA)p site for RNAs that were unspliced at the second intron. The ChpPV P5 probe, which spans the A2 acceptor site of the second intron, protected bands of 240 nts and 139 nts (Fig. 2B, lane 5), which represent unspliced and spliced RNAs at the A2 site, respectively. The ratio of RNA spliced *vs.* unspliced at the A2 site was approximately 1∶1.

### ChpPV P6 and P7 probes

**Figure 3:**
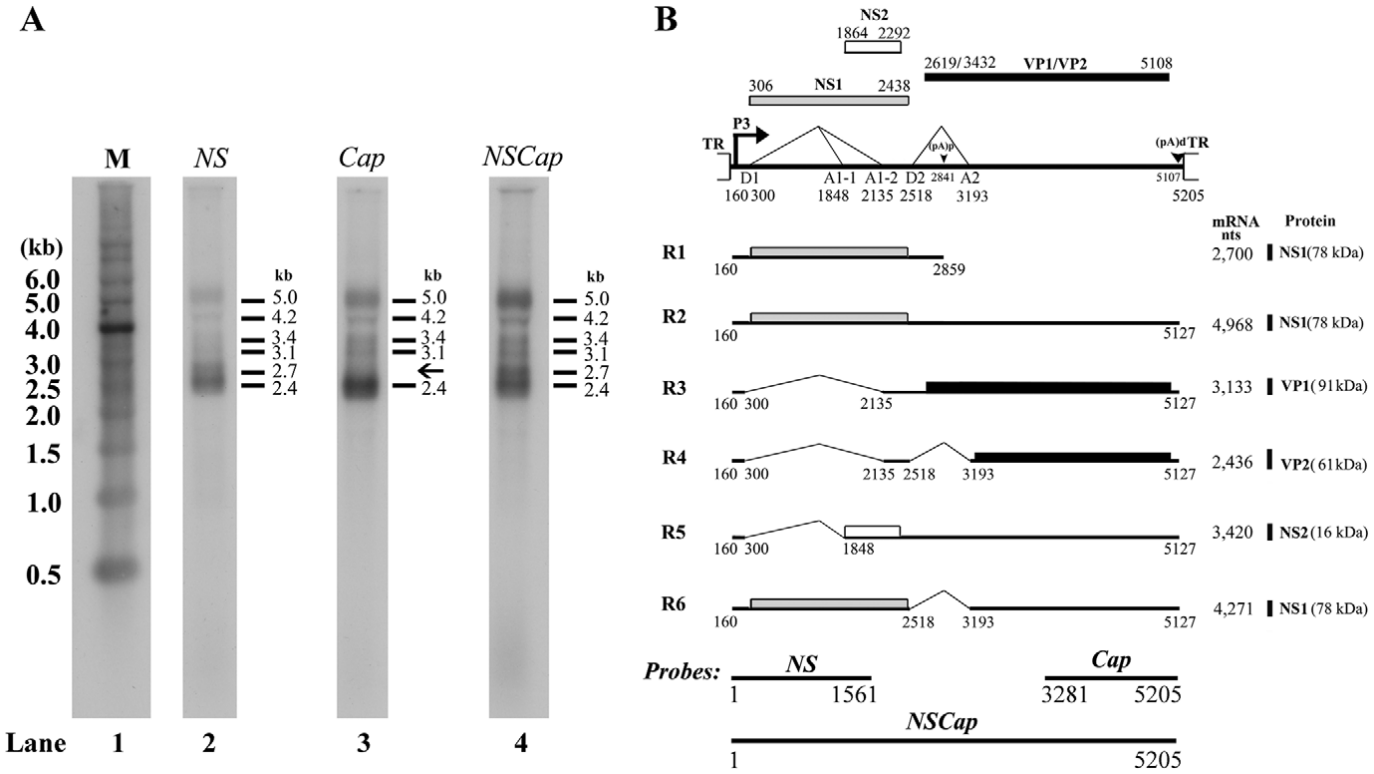
Transcription profile of ChpPV RNA. (**A**) **Northern blotting analysis**. Total RNA was isolated from COS-7 cells transfected with pC1ChpPV, and was used for Northern blotting analysis. The blots were hybridized to three ChpPV DNA probes (*NSCap*, *NS1* and *Cap*) that spanned various regions of the ChpPV genome, as diagramed at the bottom of panel B. Sizes of the RNA bands detected by each probe are indicated in kb to the right side of each lane. The RNA marker ladder is shown in lane 1. (**B**) **Transcription map of ChpPV**. The ChpPV genome is shown to scale, with transcription landmarks indicated. All of the RNA species detected in our assays are diagrammed to display their identities and respective sizes in the absence of the polyA tail. The putative ORFs that each can encode are also diagramed, and the predicted sizes (kDa) of translated proteins are indicated to the right.

The ChpPV P6 probe, which spans the distal polyadenylation site [(pA)d], was protected to yield predominantly a single band of approximately 210 nts (Fig. 2B, lane 6), enabling us to identify nt 5127 as the cleavage site at the (pA)d. The ChpPV P7 probe, which spans the putative A1-1 acceptor, was protected to generate bands of 240 nts and 153 nts (Fig. 2B, lane 8), which represent RNAs that are unspliced and spliced at the A1-1 site, respectively. The ratio of RNA that was spliced *vs.* unspliced RNA at the A1-1 site was approximately 1∶5. Alternative acceptor usage of the first intron of ChpPV, which we have confirmed in this study, was similar to that of B19V and SPV [26], [27]. However, splicing of ChpPV RNA at the A1-1 created a potential long 7.5kDa-like ORF, i.e., NS2 ORF (Fig. 3B).

### Northern blotting analysis of ChpPV RNA

Northern blotting analysis of ChpPV RNAs presented a unique profile that differed from that of other members of the genus *Erythrovirus* in that no small RNAs were processed. Hybridization of ChpPV RNA isolated from pC1ChpPV-transfected COS-7 cells with the full *NSCap* probe revealed six abundant RNA species that accumulated as bands of approximately 5.0 kb, 4.2 kb, 3.4 kb, 3.1 kb, 2.7 kb and 2.4 kb (Fig. 3A, lane 4). These six bands were also detected following hybridization of the ChpPV RNA with the *NS* probe (Fig. 3A, lane 2), indicating that all these species of ChpPV RNA were generated from a single promoter located upstream of the ChpPV genome. When using the *Cap* probe, we detected all species of ChpPV RNA except for the 2.7 kb RNA (Fig. 3A, lane 3). This suggested that the 2.7 kb RNA was polyadenylated at an internal site. Unlike in the B19V and SPV transcript profiles [26], [27], we did not detect RNA bands smaller than 2.4 kb with the three probes (Fig. 3A). This finding, together with results obtained from both RNase protection and RT-PCR analyses, suggests that a second acceptor site for the second intron in the capsid proteins-encoding region is not present, precluding the processing of small mRNAs that could encode an 11kDa-like small non-structural protein as in the B19V and SPV transcription maps [26], [27].

A transcription map of ChpPV expression in COS-7 cells was generated and is shown in Fig. 3B. Although the (pA)p site that lies in the intron between the D2 donor and the A2 acceptor was functional as assessed by RNase protection (Fig. 2B, lane 4), RNAs that were spliced at the first intron (D1 to A1-1 or A1-2) were not polyadenylated at the (pA)p. Only unspliced RNA was polyadenylated at the (pA)p and this was recognized as the R1 mRNA at 2.7 kb by both the *NS* and *NSCap* probes, but not by the *Cap* probe, in Northern blots (Fig. 3A). The R5 mRNA that was spliced from D1 to A1-1 sites and polyadenylated at the (pA)d likely encodes a small non-structural protein (NS2).

### Phylogenetic relationship of ChpPV with other parvoviruses in the subfamily *Parvovirinae*

**Figure 4:**
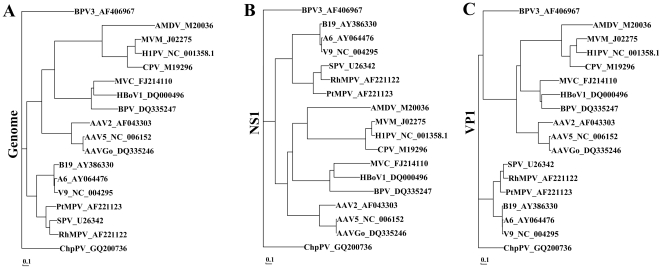
Phylogenetic analysis of ChpPV with the other 17 representative parvoviruses in each genus of *Parvovirinae*. Genome sequences (**A**), NS1 (**B**) and VP1 (**C**) amino acid sequences of representative members in each genus of the subfamily *Parvovirinae* were used to determine the neighbor-joining phylogenetic tree using BioEdit Version 7. Genbank accession numbers of the sequences used follow the name of the virus.

Since the transcription map of the ChpPV is different from those of the B19V [18], [26] and SPV [27], [28] in the genus *Erythrovirus*, which show an abundance of small RNAs produced through alternative splicing and polyadenylation [26], [27], we reexamined the phylogenetic relationship of ChpPV with other parvoviruses in the subfamily *Parvovirinae*. The new ChpPV sequence GQ200736 that has 109 nts extended from the left hand of the genome [6], and the nucleotide sequences of 17 members in the subfamily *Parvovirinae* were compared. BioEdit Version 7 (http://www.mbio.ncsu.edu/bioedit/bioedit.html) was used for sequence analysis [29]. The sequences were aligned using ClustalW (implanted in BioEdit Version 7). The phylogenetic tree was then determined by the Neighbor-Joining/UPGMA method version 3.6a2.1 (implanted in BioEdit Version 7), with the distance between nucleotide sequences indicated by the length of the branch. In addition, amino acid sequences of the ChpPV NS1 and VP1 proteins were aligned with those of the other 17 members in the subfamily *Parvovirinae*. As shown in Fig. 4, the ChpPV genome, NS1 and VP1 protein sequences are still distinct from those of the members in the genus *Erythrovirus*, though the genome and VP1 protein are relatively closer than other genera. The NS1 protein is relatively closer to that of the adeno-associated viruses (AAV) (Fig. 4B). Taken together, these results support that ChpPV does not belong to the genus *Erythrovirus*.

### The ChpPV NS1 protein is a potent inducer of apoptosis in UT7/Epo-S1 cells

**Figure 5:**
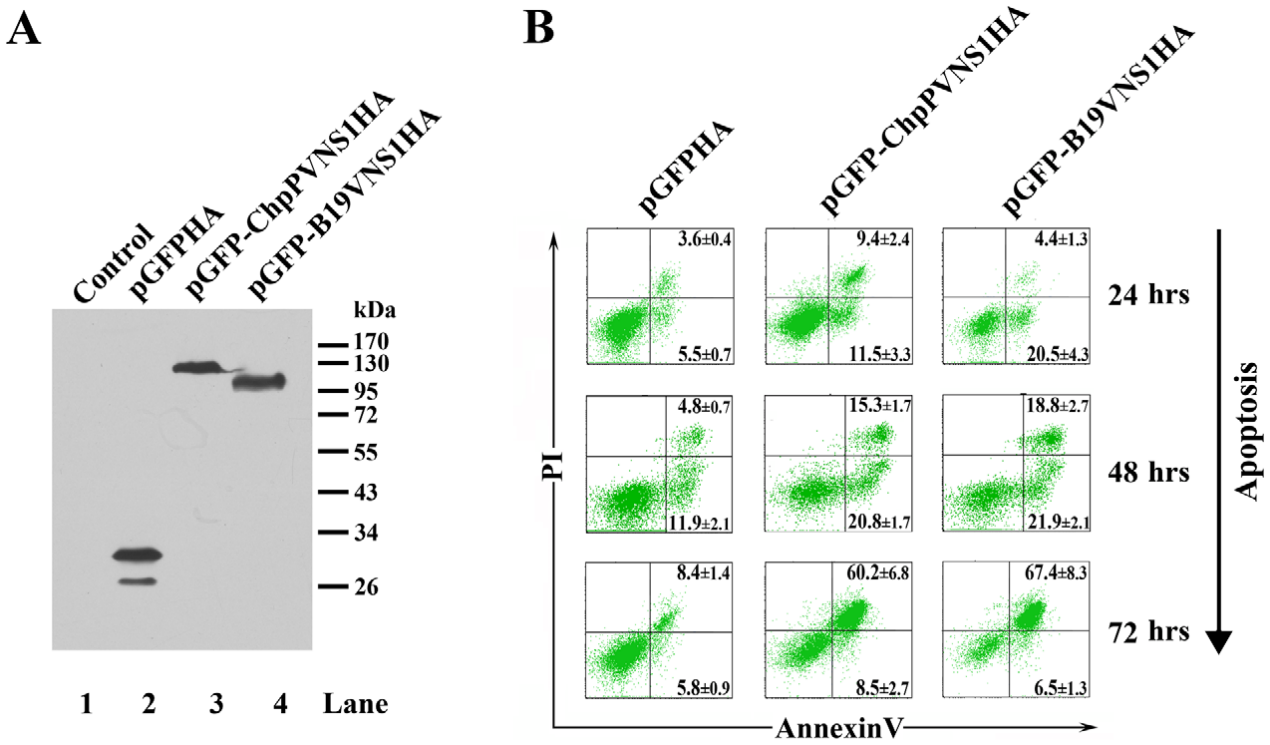
Transfection of ChpPV NS1 induces apoptosis in B19V-permissive cells. UT7/Epo-S1 cells were transfected with pGFPHA [35] (as negative control), pGFP-B19VNS1HA (positive control), and pGFP-ChpPVNS1HA. (**A**) Cells were harvested at 24 hrs posttransfection and subjected to Western blotting analysis using an anti-HA monoclonal antibody (Sigma, Clone HA-7). Untransfected cells were used as control (lane 1). A molecular marker is shown to the right with sizes in kDa. (**B**) Cells were stained with AnnexinV and propidium iodide (PI) at 24, 48 and 72 hrs posttransfection, respectively, and were then subjected to flow cytometry analysis. GFP-positive populations were gated for plotting by PI *vs.* AnnexinV staining. Numbers represent average percentage with standard deviation from three independent experiments. A representative experiment is shown. The number shown in the upper right quadrant and the number shown in the lower right quadrant in each plot are percentages of AnnexinV+/PI+ population and AnnexinV+/PI- population, respectively.

Because of the lack of the small RNAs to encode an 11kDa-like protein in the ChpPV transcription profile, we examined the proapoptotic nature of the ChpPV NS1. We transfected GFP-fused ChpPV NS1 construct into UT7/Epo-S1 cells. GFP-fused B19V NS1 and GFP alone were used as positive and negative controls, respectively. Transfected cells were confirmed to express the full length GFP-fused ChpPV and B19V NS1 at 24 hrs post transfection (Fig. 5A, lanes 3&4). Transfected cells were co-stained alive with AnnexinV and PI, followed by flow cytometry analysis. At 24 hrs post transfection, results showed that the ChpPV NS1 protein behaved much like B19V NS1, inducing a significantly higher percentage of AnnexinV+ cells in the GFP-ChpPVNS1-expressing population (GFP+) compared with the GFP control (Fig. 5B, 24 hrs). In the GFP+ population, transfection of GFP-ChpPVNS1 and GFP-B19VNS1 induced an AnnexinV+/PI- population of 11.5% and 20.5%, respectively, which represent cells at early stages of apoptosis, and induced an AnnexinV+/PI+ population of 9.4% and 4.4%, respectively, which represent cells at late stages of apoptosis. At 48 and 72 hrs post transfection, the percentages of AnnexinV+/PI+ population in GFP-ChpPVNS1- and GFP-B19VNS1-trasnfected cells (GFP+) were consistently increased as a consequence of progressing apoptosis, which are significantly higher than those of the GFP control (Fig. 5B, 48&72 hrs). We observed an overall ∼15% of AnnexinV+ population in the GFP control group at 48 and 72 hrs post transfection, which, presumably, was induced by the electroporation and the GFP. Taken together, these results suggest that, similar to the B19V NS1, the novel ChpPV NS1 protein is also an apoptosis inducer.

## Discussion

We have characterized the detailed transcription profile of ChpPV by transfecting a nearly-full length clone in COS-7 cells, which shows unique features from those of the members of the genus *Erythrovirus*. All ChpPV RNAs were generated from a single pre-mRNA that was transcribed from only one promoter, which was located at the beginning of the left-hand of the genome. ChpPV pre-mRNA contains two introns and two polyadenylation sites, and in this respect is similar to the pre-mRNA of B19V and SPV [18], [26]–[28]. However, unlike members in the genus *Erythrovirus*, ChpPV did not generate small RNA transcripts following transfection of COS-7 cells, suggesting that a small protein, i.e., 11 kDa in B19V, is not expressed during virus infection. Moreover, in all the four viruses in the genus *Erythrovirus*, including B19V, SPV, PtMPV and RhMPV, an 11kDa-like ORF is present and overlaps with the C-terminus of the VP1/2 ORF [30]. The amino acid sequence alignment of the 11kDa-like proteins among the four members shows a great similarity at the C-terminus, indicating that the 11kDa-like protein is likely an essential and conserved non-structural protein in the genus *Erythrovirus*. However, no ORF longer than 25 amino acids is present in a different frame at the C-terminus of the ChpPV VP1/2 ORF [30].

The transcription profiles of B19V and SPV characterized by transfection of their genomes in COS-7 cells were similar to those identified during virus infection of permissive cells [18], [27], [31], [32]. We believe that the ChpPV transcription profile characterized in COS-7 cells reflects the profile expressed during virus infection. Therefore, the unique transcription profile of ChpPV that we have characterized distinguishes the virus from the members in the genus *Erythrovirus*.

B19V infection induces cell death through an apoptotic pathway, in both primary erythroid cells and B19V semi-permissive cell lines, and this cell death has been attributed to the NS1 protein [8], [13]. However, we have recently discovered that the small non-structural protein 11 kDa, which is expressed abundantly, induces apoptosis during B19V infection [14]. In contrast, small RNAs that encode an 11kDa-like protein were not generated by transfecting the ChpPV genome into COS-7 cells (Fig. 3A). Nevertheless, our results have confirmed that the divergent ChpPV NS1 protein possesses a similar potency in inducing apoptosis in B19V-permissive cells, indicating that the ChpPV NS1 protein may play a key role in viral pathogenesis as the 11kDa-like protein may not be expressed. The NS1 protein of several parvoviruses in the genera *Erythrovirus*, *Parvovirus and Dependovirus* induces cytotoxicity (apoptosis) of infected cells [8]–[11]. In addition, our characterization of the ChpPV transcription profile suggests that a small non-structural protein (NS2) could be expressed from a different reading frame that lies within the NS1-encoding sequence, similar to the way in which B19V produces the 7.5 kDa protein [33], *Bocavirus* minute virus of canines (MVC) expresses the NP1 protein [23], and *Parvovirus* minute virus of mice (MVM) produces the C-terminus of the NS2 protein [34].

Although the International Committee on Taxonomy of Viruses (ICTV) has tentatively classified ChpPV as a species in the genus *Erythrovirus* [1], our phylogenetic analysis of ChpPV with other parvoviruses in the subfamily *Parvovirinae* indicates that ChpPV does not belong to any present genus in *Parvovirinae*. Together with the unique expression strategy of ChpPV that combines features from the genera *Parvovirus*, *Bocavirus* and *Erythrovirus*, this finding leads us to conclude that ChpPV may represent a new genus in the subfamily *Parvovirinae*.
